# The place of hyperbaric oxygen therapy and ozone therapy in sudden hearing loss^☆^

**DOI:** 10.1016/j.bjorl.2017.03.007

**Published:** 2017-04-08

**Authors:** Serkan Ergözen

**Affiliations:** ASAL Hyperbaric Oxygen Treatment Center, Yunus Emre Mah, Ankara, Turkey

Dear Editor,

I’ve read the valuable article entitled “The place of hyperbaric oxygen therapy and ozone therapy in sudden hearing loss” by Gülin Ergun Taşdöven et al.^1^ In this article, the authors mentioned that the cost of Hyperbaric Oxygen Therapy (HBOT) for Idiopathic Sudden Sensorineural Hearing Loss (ISSNHL) was not met by any insurance company. However, this information is incorrect, so I would like to give some information about the payment list of the Government Social Security System (GSSS) in Turkey.

An indication list of payments covered by GSSS is published every year in Turkey. The inclusion criteria for payments are also included on the list.

ISSNHL is one of the indications which has been paid for by the Turkish social security system for years, including 2010. In 2010, the GSSS criteria was “The patient must begin hyperbaric oxygen therapy within one month of the diagnosis of ISSNHL. These patients are administered a weekly audiological test. If the hearing gain is <10 dB (pure tone average) after two weeks of treatment (10 sessions), subsequent sessions will not be paid for but if the hearing gain is equal to or higher than 10 dB, 30 more sessions can be paid for by GSSS”.^2^ The criteria in use since 2012 is “ISSNHL must be diagnosed by audiological test in the last 30 days. If the hearing gain is <20 dB (pure tone average) after the 20th session of treatment, subsequent sessions will not be paid for but if the hearing gain is equal to or higher than 20 dB, 20 more sessions will be paid for by GSSS”.^3^

In addition to the GSSS payment, private HBOT clinics in Turkey can request an extra fee for each session, which may vary between 0 and 15 U.S. dollars. For example, patients pay only 2.5 USD extra fee for each session in our clinic.

Apart from ISSNHL, the GSSS and some private insurance companies pay for HBOT for the indications of decompression sickness, air or gas embolism, carbonmonoxide poisoning, cyanide poisoning, acute smoke inhalation, gas gangrene, necrotizing soft tissue infections, crush injuries, compartment syndrome, acute traumatic ischemia, diabetic and non-diabetic chronic wounds, chronic refractory osteomyelitis, radiation necrosis, compromised flaps and grafts, thermal injuries, brain abscess, anoxic encephalopathy, retinal artery occlusion, acute osteomyelitis of skull–sternum–vertebrate, and avascular necrosis of bone.^3^

## Conflicts of interest

The author declares no conflicts of interest.
